# Supplementary material for: Approaching diamond’s theoretical elasticity and strength limits
Source: Nat Commun. 2019 Dec 4;10:5533. doi: 10.1038/s41467-019-13378-w (PMC6892892; doi:10.1038/s41467-019-13378-w)
Supplement: Supplementary file 1 — Supplementary Information [file 41467_2019_13378_MOESM1_ESM.docx]

**Supplementary Information**

**Approaching diamond’s theoretical elasticity and strength limits**

Anmin Nie^1#^, Yeqiang Bu^2,3#^, Penghui Li^1#^, Yizhi Zhang^2,3^, Tianye Jin^4^, Jiabin Liu^2,3^, Zhang Su^1^, Yanbin Wang^5^, Julong He^1^, Zhongyuan Liu^1^, Hongtao Wang^2,3*^, Yongjun Tian^1*^, and Wei Yang^2,3^

*^1^Center for High Pressure Science, State Key Laboratory of Metastable Materials Science and Technology, Yanshan University, Qinhuangdao 066004, China*

*^2^Center for X-mechanics, Zhejiang University, Hangzhou 310027, China*

*^3^Institute of Applied Mechanics, Zhejiang University, Hangzhou 310027, China*

*^4^Center for Precision Engineering, Harbin Institute of Technology, Harbin 150001, China*

*^5^Center for Advanced Radiation Sources, University of Chicago, Chicago, Illinois 60439, USA*

*^#^**These authors contributed equally to the work.*

*To whom correspondence should be addressed. E-mails:* [htw@zju.edu.cn](mailto:htw@zju.edu.cn) *(Hongtao Wang), and* [fhcl@ysu.edu.cn](mailto:fhcl@ysu.edu.cn) *(Yongjun Tian)*


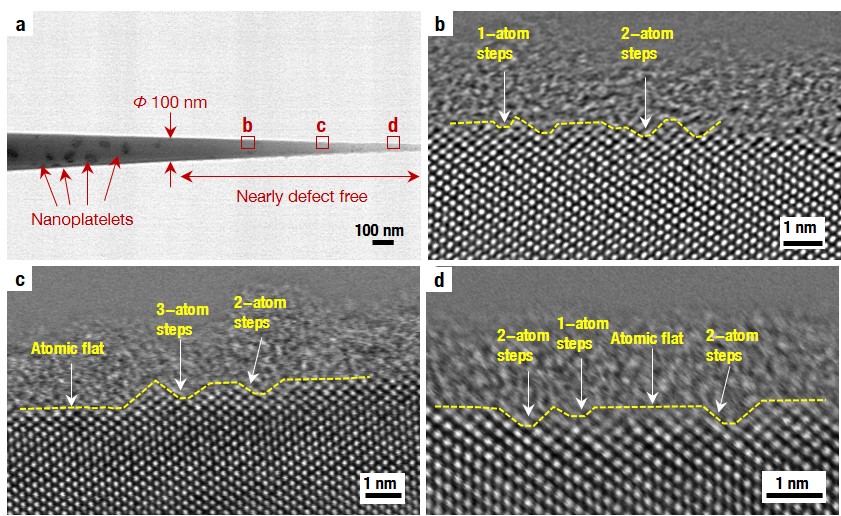


**Supplementary Figure 1 | Characterization of a typical diamond nano needle. a**, TEM image revealing size and distribution of the typical {001} platelet defects in type-Ia diamond. **b-d**, ABF-STEM images depicting the atomic scale sample surface structures at locations indicated in a. All surface structures are characterized by atomic flat lattice facets separated by sparsely distributed 1-3 atom steps, which are invariable to nano needle diameters.


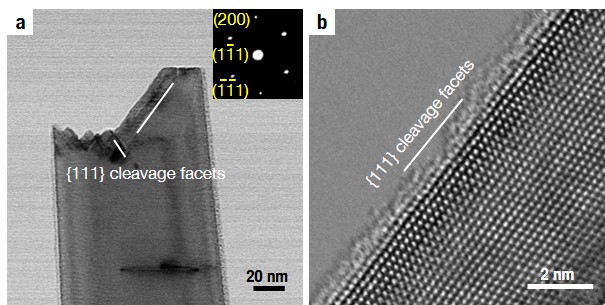


**Supplementary Figure 2 | Characterization of the fracture surfaces of a <100>-oriented diamond nanoneedle. a,** The low-magnification ABF-STEM image showing the morphology of the fracture surface. The lines are in parallel to {111} planes. Inset to a is the SEAD pattern of the diamond nanoneedle. **b**, Atomic resolved ABF-STEM image showing the atomic scale structure of a {111} cleavage facet.


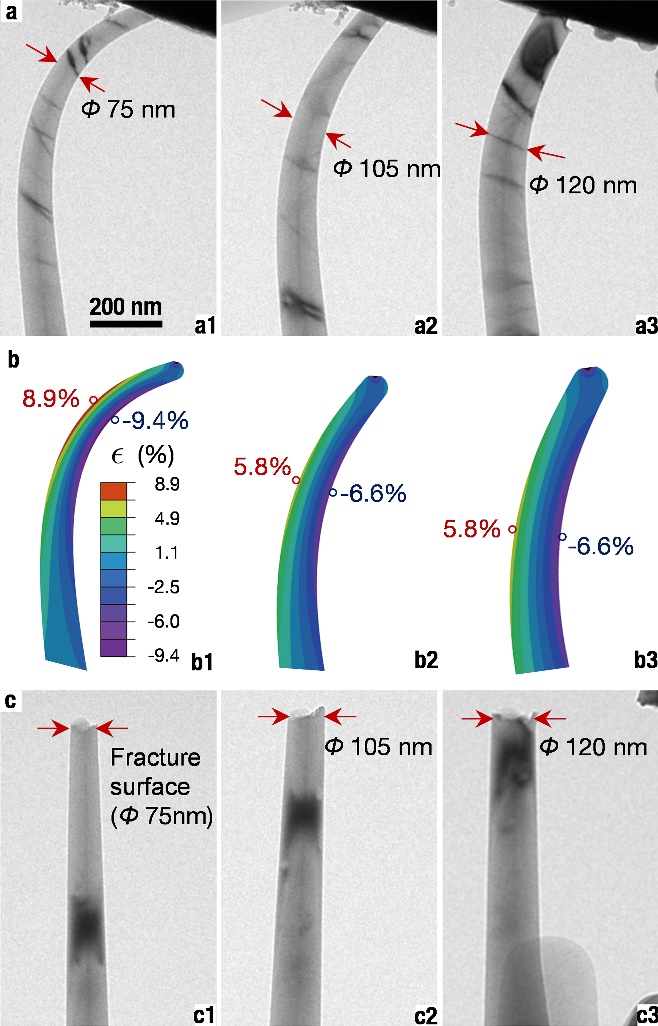


**Supplementary Figure 3 | Breaking sequence of a <110>-oriented diamond nanoneedle. a,** Snapshots from Movie S5 capturing the maximum deformation immediately before the fracture during sequentially breaking the diamond nanoneedle for its high aspect ratio geometry. The arrows indicate the subsequent fracture locations. **b**, FEM simulation reproducing the critical geometries in a and showing the maximum principle strain distribution for the nanoneedle. **c**, TEM images reveal that the corresponding fracture surfaces consist of {111} facets.


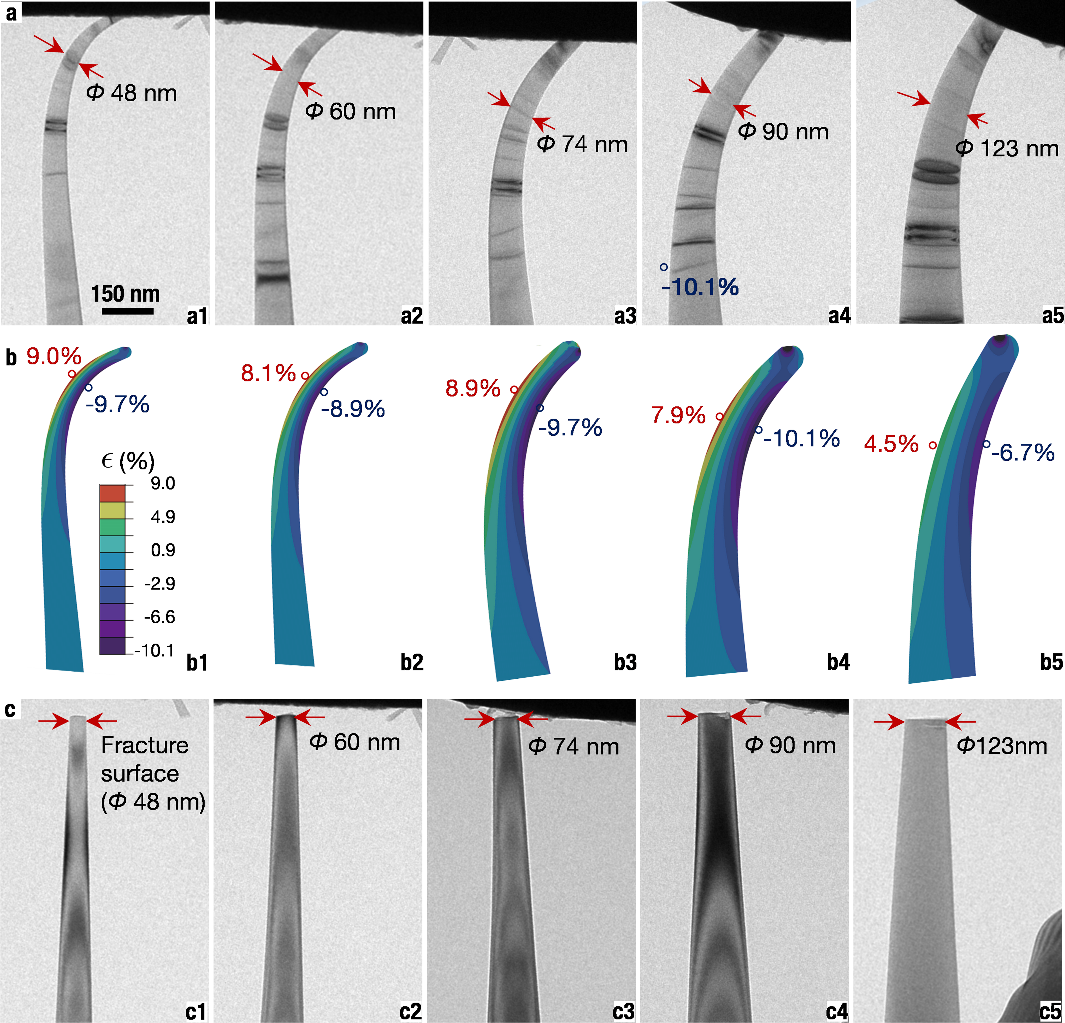


**Supplementary Figure 4 | Breaking sequence of a <111>-oriented diamond nanoneedle. a,** Snapshots from Movie S5 capturing the maximum deformation immediately before the fracture during sequentially breaking the diamond nanoneedle for its high aspect ratio geometry. The arrows indicate the subsequent fracture locations. **b**, FEM simulation reproducing the critical geometries in a and showing the maximum principle strain distribution for the nanoneedle. **c**, TEM images reveal that the corresponding fracture surfaces are the {111} plane.


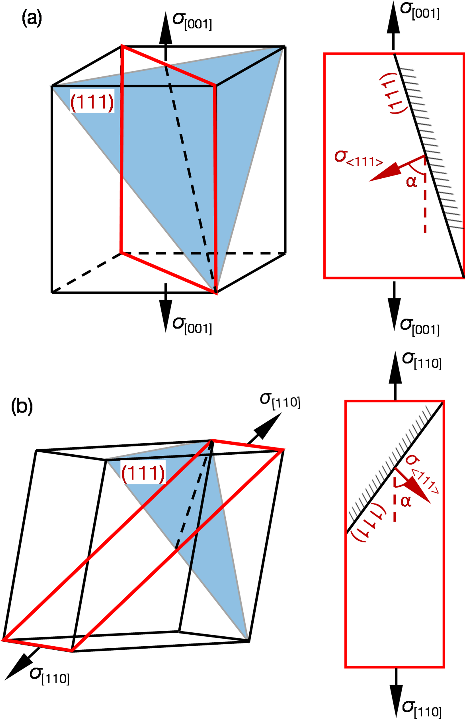


**Supplementary Figure 5 | Illustration of the ideal strength projection from (a) [100] and (b) [110] to [111]. a**, The lattice of is diamond elongated into a tetragonal structure at the large strain of 38%. The right panel of **a** is a sectional view corresponding to the plane outlined by red lines. The (111) plane is viewed along edge-on in the right panel. Operating a simple coordinate system conversion^1^, the normal stress on (111) plane (*σ*_[111]_) could be calculated as *σ*_[111]_ = *σ*_[001]_cos^2^*α*, $\cos\alpha=\frac{1+\varepsilon}{\sqrt{\text{1+1+（1+}\varepsilon\text{）}^{2}}}$, where *ɛ* is strain along [001] direction, *α* is the angle between [1,1,1+*ɛ*] and [0,0,1+*ɛ*] direction in the severe deformed lattice. The ideal strength approached in the <100>-orientated diamond nanoneedles, i.e., *σ*_[001]_ = 225 GPa when the *ɛ* = 38%. the resultant stress along C-C bond, i.e., *σ*_[111]_ = 109 GPa. **b**, For the <110>-loading case, the projection process is similar with that of <100>-loading case. Projecting the the ideal strength along [110] onto [111] through the coordinate system conversion. Herein, *σ*_[111]_ = *σ*_[001]_cos^2^*α*, $\cos\alpha=\frac{\sqrt{2}\text{(1+}\varepsilon\text{)}}{\sqrt{\text{2(1+}\varepsilon)^{2}\text{+1}}}$, where ɛ is strain along [110] direction, *α* is the angle between [1+*ɛ*,1+*ɛ*,1] and [1+*ɛ*,1+*ɛ*,0] direction in the severe deformed lattice. The ideal strength approached in the <100>-orientated diamond nanoneedles, i.e., *σ*_[001]_ = 126 GPa when the *ɛ* = 26%. the resultant stress along C-C bond, i.e., *σ*_[111]_ = 95 GPa.


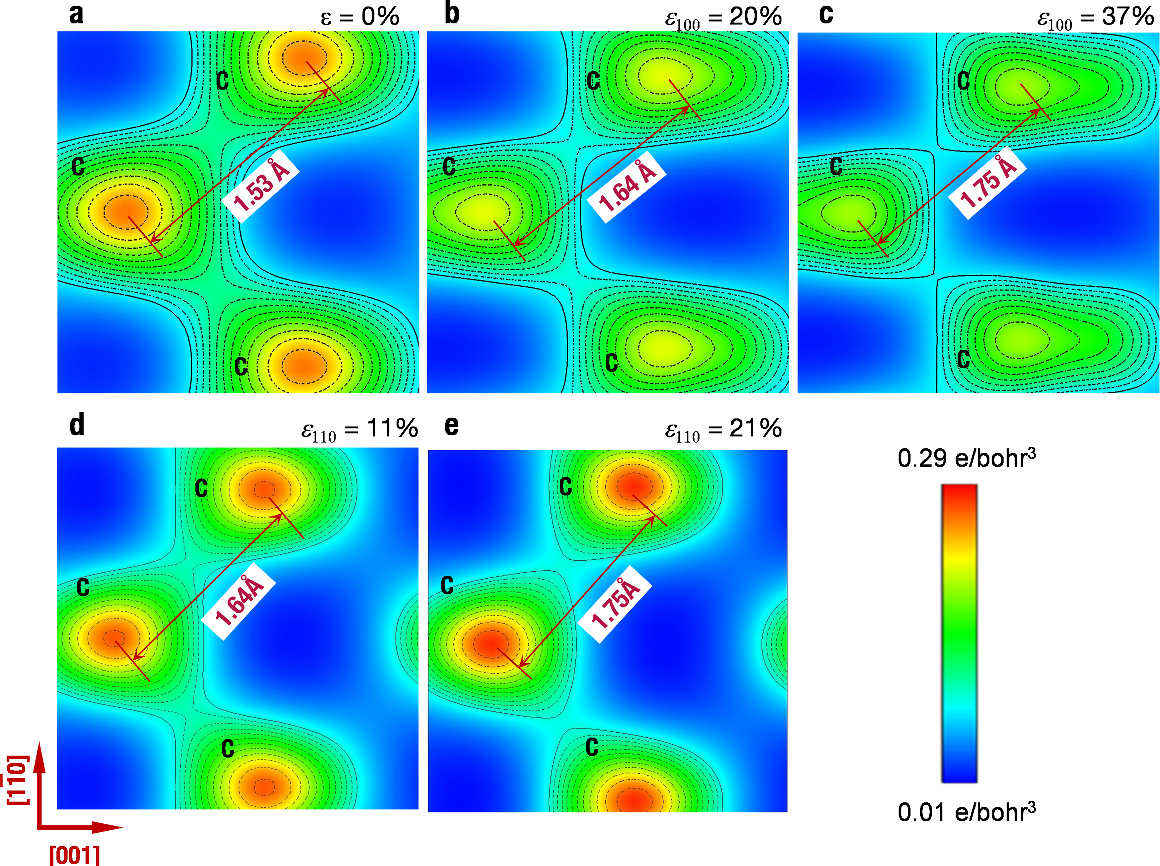


**Supplementary Figure 6 | The first principle simulations of a bulk diamond under [001] and** $[1\bar{1}0]$ **direction tension.** The contour plots of the valence charge density of the C-C bond under (**a**) strain of 0%, (**b-c**) strains of 20% and 37% on [001] direction, (**d-e**) strains of 11% and 21% on $[1\bar{1}0]$ direction.


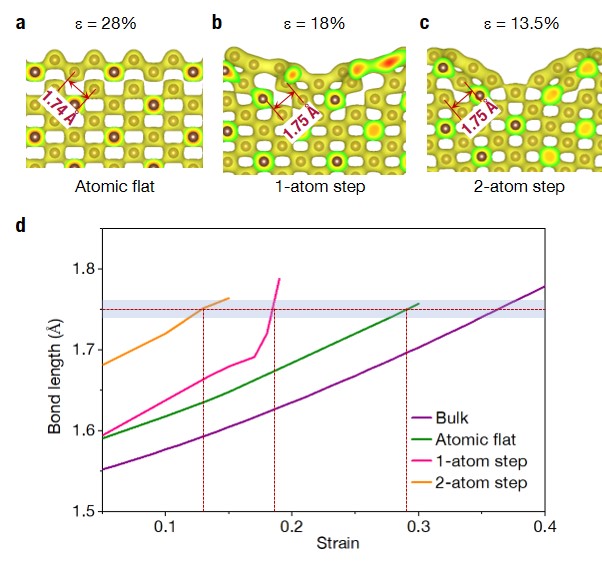


**Supplementary Figure 7 | The first principle simulations of a diamond under [100] uniaxial tension with a free surface. a-c,** Surface plots of the valence charge density at 0.165 e/Bohr^3^ of a diamond with different surface structures under [100]-direction tensile strains: **a**, atomically flat surface and *ɛ* = 28% (*σ*_[100]_ = 205 GPa); **b**, free surface with 1-atom step and *ɛ* = 18%(*σ*_[100]_ = 157 GPa) ; and **c**, free surface with 2-atom step and *ɛ* = 13.5%(*σ*_[100]_ = 125.4 GPa) . The atomic configurations are the same as Fig. 5c-e. The maximal bond lengths are denoted in the charge density plots. **d**, The corresponding bond lengths change with the uniaxial tensile strain.


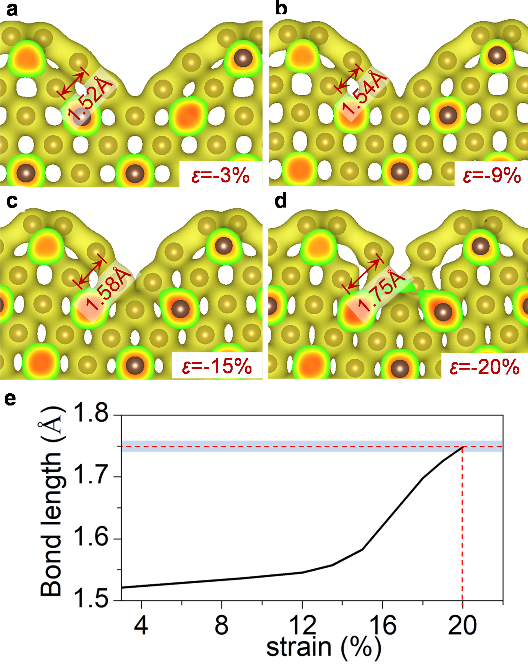


**Supplementary Figure 8 | The first principle simulations of a diamond under [100] uniaxial compression with two-step surface** **flaw. a-c**, Surface plots of the valence charge density at 0.165 e/Bohr^3^ under [100]-direction compressive strains of *ɛ* = -3%; *ɛ* = -9%; *ɛ* = -15% and *ɛ* = -20%, respectively. **e**. The maximal bond lengths denoted in the charge density plot change with the uniaxial tensile strain.


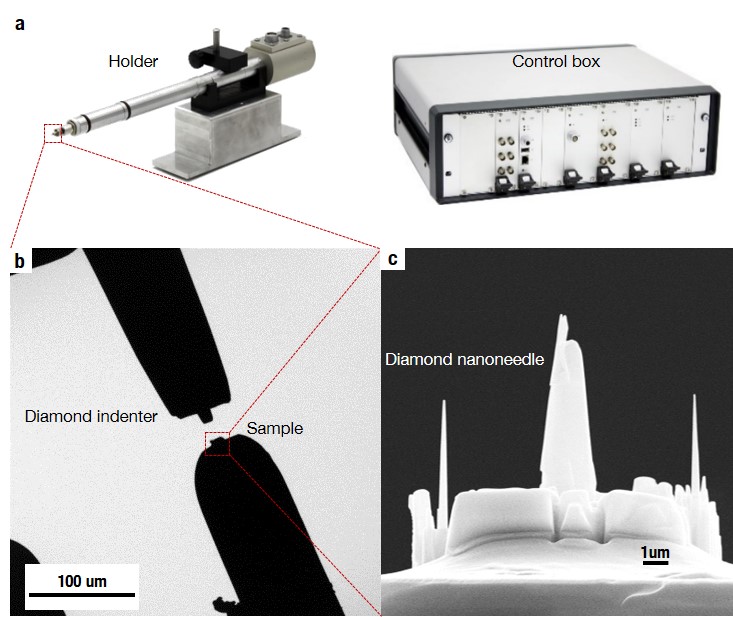


**Supplementary Figure 9 | The instrumentation and the experimental setup for the in-situ bending tests. a,** The XNano TEM holder and controller developed by the co-corresponding author Hongtao Wang’s group at the center for X-Mechanics, Zhejiang University. The images were created by our co-authors. **b**, The low magnification TEM image showing the relative position of the sample and a diamond indenter inside TEM. **c**, The SEM image showing the FIB prepared high aspect ratio diamond nanoneedles.

**Supplementary References**

1. Landau L, Lifshitz E. Theory of Elasticity, second revised and enlarger edition, Vol. 7. (ed^(eds). Pergamon press (1981).
