# Supplementary material for: Approaching diamond’s theoretical elasticity and strength limits
Source: Nat Commun. 2019 Dec 4;10:5533. doi: 10.1038/s41467-019-13378-w (PMC6892892; doi:10.1038/s41467-019-13378-w)
Supplement: Supplementary file 2 — Description of Additional Supplementary Files [file 41467_2019_13378_MOESM2_ESM.docx]

**Description of Additional Supplementary Files**

**Supplementary Movie 1** | In-situ bending test of a <100>-oriented diamond nanoneedle.

**Supplementary Movie 2** | In-situ bending test of a <100>-oriented diamond nanoneedle.

**Supplementary Movie 3** | In-situ bending test of a <100>-oriented diamond nanoneedle.

**Supplementary Movie 4** | The breaking sequence of a <100>-oriented diamond nanoneedle.

**Supplementary Movie 5** | The breaking sequence of a <110>-oriented diamond nanoneedle.

**Supplementary Movie 6** | The breaking sequence of a <111>-oriented diamond nanoneedle.
